# Cold Atmospheric Plasma-Activated Media Improve Paclitaxel Efficacy on Breast Cancer Cells in a Combined Treatment Model

**DOI:** 10.3390/cimb44050135

**Published:** 2022-04-30

**Authors:** Cosmin-Teodor Mihai, Ilarion Mihaila, Maria Antoanela Pasare, Robert Mihai Pintilie, Mitica Ciorpac, Ionut Topala

**Affiliations:** 1 Advanced Research and Development Center for Experimental Medicine (CEMEX), Grigore T. Popa University of Medicine and Pharmacy of Iasi, 700115 Iasi, Romania; antoanela.pasare@gmail.com (M.A.P.); robert.pintilie95@gmail.com (R.M.P.); mitica.ciorpac@umfiasi.ro (M.C.); 2 Integrated Centre of Environmental Science Studies in the North-Eastern Development Region (CERNESIM), Alexandru Ioan Cuza University of Iasi, 11 Carol I Blvd., 700506 Iasi, Romania; ilarion.mihaila@gmail.com; 3 Iasi Plasma Advanced Research Centre (IPARC), Faculty of Physics, Alexandru Ioan Cuza University of Iasi, 11 Carol I blvd., 700506 Iasi, Romania

**Keywords:** breast cancer cell, plasma-activated media, PTX, combined therapy, cell cytotoxicity, apoptosis, DNA damage

## Abstract

**Simple Summary:**

Breast cancer is the most commonly diagnosed cancer and the leading cause of cancer mortality in females, with chemotherapy currently the only adjunctive therapy. However, the disadvantages of chemotherapy are represented by toxicity/side effects, and for many women the benefit is uncertain. Cold atmospheric plasma in in vitro and in vivo experiments proved that it is able to selectively inhibit the growth of cancer cells in various cancer types, with less deleterious side effects. Current guidelines include paclitaxel as a chemotherapy of choice for various types of breast cancers. The aim of the study was to evaluate in vitro, on human breast cancer cell lines, a plasma-activated media as a cytotoxic agent and as an associative agent in a combined treatment for breast cancer with paclitaxel. The collected data indicated that the plasma-activated media amplified the cytotoxic effect of paclitaxel.

**Abstract:**

The use of plasma-activated media (PAM), an alternative to direct delivery of cold atmospheric plasma to cancer cells, has recently gained interest in the plasma medicine field. Paclitaxel (PTX) is used as a chemotherapy of choice for various types of breast cancers, which is the leading cause of mortality in females due to cancer. In this study, we evaluated an alternative way to improve anti-cancerous efficiency of PTX by association with PAM, the ultimate achievement being a better outcome in killing tumoral cells at smaller doses of PTX. MCF-7 and MDA-MB-231 cell lines were used, and the outcome was measured by cell viability (MTT assay), the survival rate (clonogenic assay), apoptosis occurrence, and genotoxicity (COMET assay). Treatment consisted of the use of PAM in combination with under IC50 doses of PTX in short- and long-term models. The experimental data showed that PAM had the capacity to improve PTX’s cytotoxicity, as viability of the breast cancer cells dropped, an effect maintained in long-term experiments. A higher frequency of apoptotic, dead cells, and DNA fragmentation was registered in cells treated with the combined treatment as compared with those treated only with PT. Overall, PAM had the capacity to amplify the anti-cancerous effect of PTX.

## 1. Introduction

Breast cancer is the most commonly diagnosed cancer and the leading cause of cancer mortality in females [1]. According to IARC, breast cancer has surpassed lung cancer, and is now the number one cause of cancer worldwide. Some of the most relevant risk factors for this type of cancer are: age, early menarche and late menopause, obesity and lifestyle related factors, and hormone replacement therapy [2]. Treatment efficiency and survival prognostics are dependent on the identification of breast cancer subtypes based on the presence of estrogen receptor (ER), progesterone receptor (PR), and human epidermal growth factor receptor type 2 (HER2). Triple-negative (TN) patients lack all three. Compared to other subtypes, triple-negative breast cancers (TNBC) are more frequent and aggressive, with high potential to metastasize and less options of targeted treatment.

Chemotherapy is currently the only adjunctive therapy and is the treatment of choice for patients diagnosed in the late stages of locally advanced and metastatic cancers [3]. However, its disadvantages are represented by toxicity/side effects and the fact that some tumor cells can still maintain viability after chemotherapy. For many women, the benefit of chemotherapy is uncertain. Therefore, establishing new therapies for TNBC with few side effects is necessary [4,5].

In vitro and in vivo experimental data demonstrated that cold atmospheric plasma (CAP) is able to selectively inhibit the growth of cancer cells in various cancer types, when compared with the normal counterparts [6,7,8,9,10,11,12]. By generating reactive oxygen and nitrogen species (RONS), CAP induces a direct effect on cells and acts as an intracellular signaling trigger [7]. Immediate effects are the occurrence of apoptosis [13,14,15,16,17,18,19] and cell cycle arrest [20,21]. In addition, CAP determined the detachment of tumoral cells from the extracellular matrix, decreased migration velocity of cells [22], as well as inhibited the metastatic processes [14]. By increasing the number of the cells in the S phase of the cell cycle, their susceptibility to the CAP or another cytostatic action is higher, being possible the destruction of a larger population of cancerous cells [20]. Another important trait of plasma treatment is that the treatment has delayed effects, with the cell viability constantly reducing after the treatment with plasma-activated media [23].

From this point of view, CAP is a novel and promising alternative agent for classical oncology therapies either as monotherapy or as an adjuvant [24,25,26,27]. 

Identification of changes in the media composition by exposure to CAP was acquired by using jet plasma as the plasma source, as well as the less often used dielectric barrier discharge plasma sources. RONS could be short-lived transient species (peroxynitrite ONOO-, hydroxyl -OH) and long-lived species (ozone O_3_, hydrogen peroxide H_2_O_2_, nitric acid HNO_3_, nitrous acid HNO_2_) [23]. In addition, plasma-produced reactive species vary in lifetime, ranging from nanoseconds to hours [28]. The primary RONS, such as ^•^OH, ^•^NO, H_2_O_2_, O, and O_3_, are produced in gas phase plasma, and when in contact with the liquid undergo transformations. Secondary RONS (^•^OH and ^•^NO2 from HOONO and HNO_2_, and HNO_3_ from ^•^NO and ^•^NO_2_) result from interaction or degradation of primary RONS with each other or with molecules from the media [29,30]. Moreover, dependent on the plasma characteristics, biological effects could be more or less strong, correlated with the amount or proportion between different types of RONS. Therefore, in an experimental setup using positive and negative pulse plasma jet, the levels of H_2_O_2_, O_2_^−^, OH, and ONOO^−^ in medium from the positive pulse plasma jet were higher than that from the negative pulse plasma jet, while the negative plasma jet generated higher levels of the aqueous NO_2_^−^ and NO_3_^−^. Furthermore, the impact on apoptosis and cell viability was more pronounced in the case of positive plasma [31].

In addition to direct treatment, in indirect treatment, the effects of plasma are attributed to long-lived molecular and ionic chemical species, which remain in solution after CAP treatment, such as H_2_O_2_, NO_2_^−^, and NO_3_^−^. Similar antiproliferative effects were obtained in the case of multicellular tumor spheroids (MCTS), when were treated with preconditioned media or by immersion of MCTS in culture media exposed to low temperature plasma, suggesting the persistence of the long-lived RONS and their deleterious effects on cancer cells. In addition, the antiproliferative effect was abolished by use of ROS scavengers [30]. Similar results were obtained on colon adenocarcinoma multicellular tumor spheroids in which H_2_O_2_ was identified as responsible for DNA damage whilst reactive nitrogen species may have interfered with the proliferation process. These effects were registered even in the condition of long-term storage of the activated media at +4 °C or −80 °C, due to the ability of H_2_O_2_ to remain stable at these temperatures and to maintain its chemical composition [32,33].

Medium composition also influences the stability of different RONS and their interaction with other biomolecules, generating oxidized products. This combination of long-lived RONS and oxidized biological compounds decreases the essential nutrient deposits and finally induces cytotoxicity [34]. Exposure of media to plasma jets did not change the protein components of the medium, but the pH and electrical conductivity tended to increase with longer irradiation time. In the case of Dulbecco’s Modified Eagle Medium (DMEM) supplemented or not with fetal bovine serum (FBS), a decline in UV transmittivity was registered, suggesting that longer irradiation is accompanied by changes in culture medium components which may be responsible for cell death [35].

A less explored application of CAP is its use in combined therapy for breast cancer. PTX is a natural alkaloid that is highly lipophilic and acts as a cytoskeletal drug, targeting tubulin [36]. However, unlike vinca alkaloids, PTX causes polymerization of tubulin, which leads to greatly stable and dysfunctional microtubules. The antineoplastic activity is notably important for breast cancer, epithelial ovarian cancer, and colon, head, and non-small cell lung carcinoma [37]. Current guidelines include taxanes (e.g., PTX) as a chemotherapy of choice for various types of breast cancers [38]. Although taxanes have been proven to be the most active of agents against metastatic breast cancer, their use can be limited by side effects such as hypersensitivity reactions and hematological, cardiac, and neurological toxicity [39]. Cancer cells can escape PTX toxicity by changing microtubule physiology, or by developing efflux pumps and drug metabolism [40]. Park et al. showed that CAP has the potential to restore PTX sensitivity to resistant breast cancer cells [41].

Therefore, we investigated the efficiency of CAP with a PTX combined treatment against MCF-7 and MDA-MB-231 cell lines through its impact on cell viability, apoptosis, cell motility, and genotoxicity. CAP was not administered as a direct treatment, but as plasma-activated media (PAM), because PAM have been found to kill cancer cells as effectively as direct treatment [42]. In addition, PAM has the advantage of being stored and can be injected into tissues [43], easing manipulation, administration, and also its use in combination with other drugs. To our knowledge, the association of PTX with PAM has not yet been evaluated. 

Our study was focused on the evaluation of the effects of PAM applied in conjunction with PTX on the treatment of breast cancer cells MCF-7 (double positive) and MDA-MB-231 (TNBC). We evaluated whether PAM could enhance the effect of PTX, and the outcomes of the combined treatment were represented by cell viability, apoptosis, cell migration, and DNA damage.

## 2. Materials and Methods

*Cell lines.* MCF-7 (ATCC, HTB-22) and MDA-MB-231 (ATCC, CRM-HTB-26) cell lines were maintained in DMEM (Dulbecco’s Modified Eagle Medium, Biochrom AG, Berlin, Germany), supplemented with 10% FSB (fetal bovine serum, Sigma, Steinheim, Germany), 100 IU/mL penicillin (Biochrom AG, Berlin, Germany), 100 µg/mL streptomycin (Biochrom AG, Berlin, Germany), at 37 °C, in a humidified atmosphere of 5% CO_2_ in air. The cell lines were a cordial donation of professor Charalambos Anastassiou from University of Cyprus.

**Figure 1:**
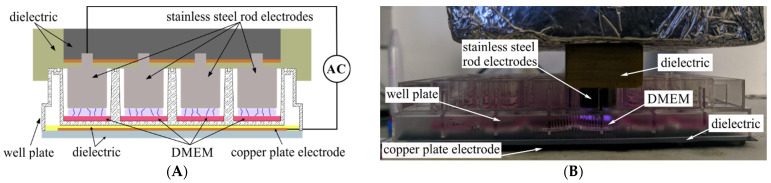
The schema (**A**) and working setup (**B**) for air DBD plasma experimental exposure system for DMEM activation.

*Generation of plasma-activated media (PAM).* We selected the DBD (dielectric barrier discharge) in air as an efficient technique to generate pulsed cold plasma at atmospheric pressure, directly into an entire column of 24-well cell culture plates. This ensures fast, safe, and efficient activation of water, buffers, and media used in biology and medicine laboratories [44]. Details of the experimental setup and a picture of the plasma region during media activation are presented in Figure 1. The cross section (Figure 1a) allows to spot the 4 stainless steel rod electrodes, and the 3 mm gap between each rod and the bottom of each well. A copper back electrode (1.5 mm tick plate) was used to mount the 24-well plate. Two dielectric layers, the plate itself and a supplementary sheet of silicon rubber, were used to eliminate any remaining air gaps. Before any experiment activation, DMEM media without FBS was transferred in all wells of a selected column, using 160 μL volume for each well.

A laboratory-made high voltage ac power supply composed of a variable autotransformer and the double sinusoidal waveform output of a neon transformer was used to break down the air gap inside the wells. Constant electrical excitation parameters (10 kV peak-to-peak, 28 kHz) were used in this study and the exposure duration was variable: 15 s (PAM 15 s), 30 s (PAM 30 s), or 60 s (PAM 60 s).

Immediately after exposure, the plasma-activated media from all wells exposed in similar conditions were collected into sterile plastic containers. The temperature inside the medium, evaluated after, was not higher than 40 °C. The procedure was repeated using different columns, to avoid heat accumulation, until a desired volume of PAM was produced.

*Cell viability assay.* Evaluation of the cells’ viability was based on MTT assay. Briefly, cells were seeded in 96-well plates, at a density of 5 × 10^3^ cells/well, and allowed to attach and grow overnight. Treatment with PAM was applied for 20 min using 50 µL of PAM, and when incubation time expired plasma medium was removed and changed with fresh DMEM supplemented with 10% FBS. PTX was added to cell cultures in doses of 0.1 µM, 0.01 µM, and 0.001 µM, either direct to well, in the case of single treatment, or after PAM treatment, in the case of the combined treatment. After the treatment period (24 and 48 h) expired, the cells were washed and covered with 100 μL of fresh DMEM 10% FBS. An amount of 10 μL of MTT (5 mg/mL) was added in medium and cells were incubated for 3 h. To solve the formed formazan, DMSO (dimethyl sulfoxide, Merck, Darmstadt, Germany) was used and the absorbance was recorded at 570 nm. Cellular viability (%) was calculated according to the formula: % cell viability = [Absorbance]sample/[Absorbance]control × 100.

*Apoptosis assay.* Apoptosis evaluation was performed accordingly to Ribble, D. et al. [45] with modifications. Cells were allowed to grow on clean cover slides on the bottom of 24-well plates, at a density of 1 × 10^4^ cells/well. After PAM, PTX, or combined treatment, cells were returned into the incubator and allowed to grow another 6 h. After the treatment period was over, the slides were carefully removed and washed with phosphate-buffered saline (PBS), double stained with ethidium bromide/acridine orange (100 μg/mL acridine orange and 100 μg/mL ethidium bromide in PBS), placed on a microscope slide, and were analyzed using a fluorescence microscope (Olympus BX41 microscope) and pictures of slides were captured. Scoring of the cells was based on the following criteria: live cells—normal green nucleus; apoptotic cells, including early apoptotic cells—bright green nucleus with condensed or fragmented chromatin, and late apoptotic cells—condensed and fragmented orange chromatin; dead cells—structurally normal orange nucleus.

*Clonogenic assay.* The test was performed according to Franken et al. [46]. Briefly, cells were seeded in 6-well plates (2 × 10^5^ cells/well) and allowed to grow overnight. Next day, the medium was discarded and in every well was added 1 mL of activated medium (15 and 30 s), but none in the control and PTX wells. After 20 min the medium was replaced with fresh DMEM or medium supplemented with 0.001 µM PTX, and cells were incubated for another 24 h. A day after the treatment, cells were detached by trypsinization, counted with a hemocytometer, and seeded in 12-well plates (300 cells/well); every 2 days the medium was replaced with fresh DMEM 10% FBS. After colonies were formed in the control group (over 50 cells/colony), colonies were washed with PBS, fixed with paraformaldehyde 4% (PFA), and stained with 0.5% crystal violet. Colonies were counted and analyzed with FIJI software.

*Scratch assay.* Cells were seeded in 24-well plates (5 × 10^3^ cells/well) and allowed to grow overnight. With a 200 µL pipet tip the cell monolayer was scratched in a straight line in the middle of the well. Cells were washed and 1 mL of activated medium (15 and 30 s) was added to each well, except PTX wells. Subsequently, 20 min later, the medium was replaced with fresh DMEM or DMEM 0.001 µM PTX, as for the clonogenic assay. After 6 and 24 h, respectively, cells were fixed with PFA 4% and stained with 0.5% crystal violet. For each well a picture was taken, and the scratches were analyzed using Ilastik software and MRI plugin in FIJI software.

*COMET assay.* The genotoxic potential was evaluated by the alkaline single-cell gel electrophoresis assay (COMET assay) [47]. Cells were seeded in 6-well plates (3 × 10^5^ cells/well) and grown overnight. After 3 h from the treatment, cells were detached by trypsinization, and the cell suspension (5 × 10^3^ cells) was mixed with 1% low melting agarose at 37 °C and quickly poured into 1% normal melting agarose precoated slides. After agarose gelification, the slides were immersed in freshly prepared cold lysis solution and lysed overnight at 4 °C in the dark. The slides were further washed with the electrophoresis buffer three times at room temperature and placed in a horizontal gel electrophoresis tank very close to the anode. The electrophoresis was carried out at 0.6 V/cm for 25 min. The procedure was performed under dimmed light to prevent additional DNA damage. After electrophoresis, the slides were rinsed with distilled water. Comets were visualized by ethidium bromide staining (20 μg/mL, 30 s) using a fluorescence microscope (Olympus BX41 microscope). Comet scoring was performed by CometScore2 software (TriTek CometScore). The results were expressed as % head DNA and as frequency.

*Spheroids.* Cells were detached from a 25 cm plate, counted, and diluted until a cell suspension of 4 × 10^3^ cells/20 µL was obtained. On the lid of Petri dishes 20 drops of 20 µL with cells were placed, and distilled water was used to maintain humidity. After 48 h of incubation, spheroids were treated with activated medium (PAM 15 s) for 20 min or with PTX. After another 24 h, each spheroid was photographed an analyzed using FIJI software. 

## 3. Results and Discussions

### 3.1. Effects of PAM and Combined Treatment on Cell Viability

The central aim of this study was the investigation of the synergistic effects of PTX with plasma-activated media, as a new method for improvement of cancer treatment. For this investigation, three doses of PTX were selected (0.001 µM, 0.01 µM, and 0.1 µM) and two batches of PAM (30 s and 60 s exposed to DBD plasma). Our finding revealed that the combination of PTX with PAM lowered the cell viability of both cancerous cell lines.

**Figure 2:**
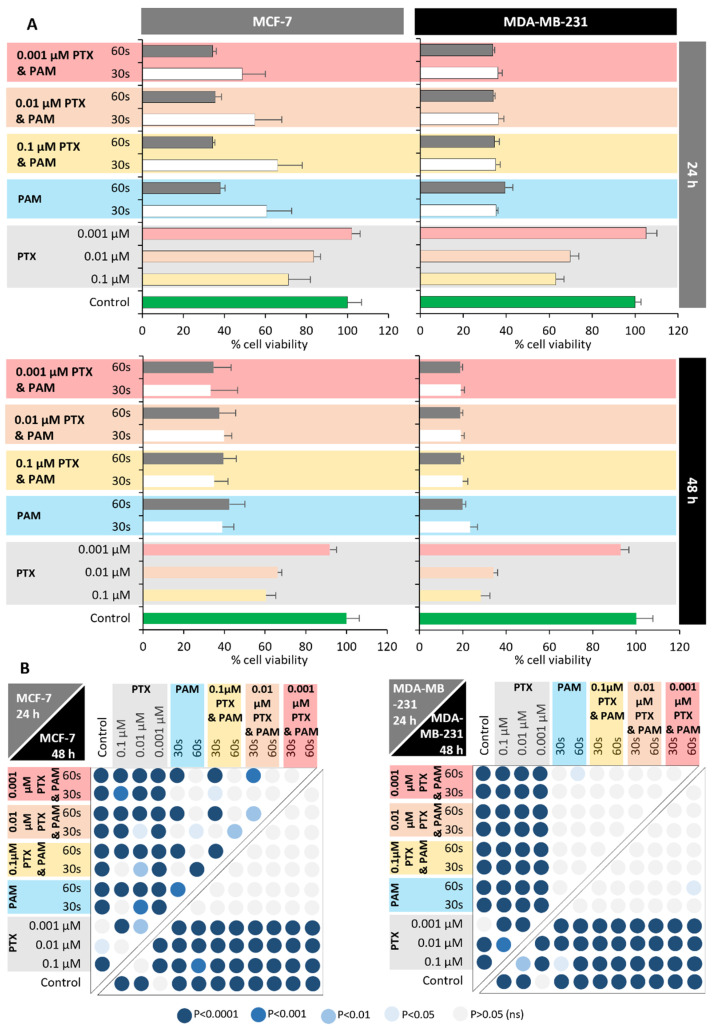
(**A**) Viability of the MCF-7 and MDA-MB-231 cell lines was determined by MTT method 24 and 48 h after the treatment with PTX in a dose ranging from 0.1 μM to 0.001 μM, PAM (30 s and 60 s), and combined treatment (PTX and PAM). PAM was obtained through media exposure to CAP for 30 s and 60 s. Treatment with PAM and incubation of the cells with PAM was 20 min. PTX was applied for 24 h and combined treatment consisted of PAM application followed by PTX incubation. Cell viability was determined by MTT assay. Control group received only normal media; PAM—treatment was applied only with plasma-activated media; PTX—treatment was applied only with PTX; PTX and PAM—combined treatment was used. Data are mean and SEM values (*n* = 8). (**B**) Statistical significance obtained by multiple comparison between different groups. Colored dots indicate the statistical significance computed after applying Tukey multiple comparison test and are in accordance with the obtained value: darker values correspond to *p* < 0.0001 and lighter ones to non-significant values.

MCF-7 and MDA-MB-231 cells were treated with PAM (30 s and 60 s, 20 min each), PTX (0.1 µM, 0.01 µM, and 0.001 µM), and with the combined treatment (PTX and PAM) in order to investigate the antitumor effect of PAM and its capacity to improve the antineoplastic effect of PTX (Figure 2A). Cell viability was determined at 24 h and 48 h after the PAM, PTX, and the combined treatment were applied.

PTX administrated to MCF-7 cells exhibited a moderate cytotoxicity directly proportional to the dosage of the chemotherapy agent. PTX reduced cell viability at mild decrements for all three doses at both time points: 24 h (102% for 0.001 µM, 83.56% for 0.01 µM, and 71.17% for 0.1 µM) and 48 h (91.89% for 0.001 µM, 66.22% for 0.01 µM, and 60.47% for 0.1 µM). The trend of direct proportionality was also observed in MDA-MB-231 cells, but with a much higher amplitude. At the 24 h point, cell viability was reduced from 105% (0.001 µM PTX) to 63.05% (0.1 µM PTX), while at 48 h the reduction was much steeper, from 92.79% (0.001 µM PTX) to 28.31% (0.1 µM PTX). Overall, the MDA-MB-231 cell line showed a higher sensitivity to PTX compared to MCF-7 cells, as registered at 24 h where the difference was up to 13.77% (0.01 µM PTX), and at 48 h with a difference up to 32.15% (0.1 µM PTX). Our findings are in accordance with Satosi et al. [48], which also reported a different level of sensitivity to PTX between MDA-MB-231 and MCF-7 cell lines. 

PAM 30 s and PAM 60 s treatments determined a significant reduction in MCF-7 and MDA-MB-231 viability at both 24 and 48 h. The MDA-MB-231 cell line was more susceptible to plasma-activated media as registered at 48 h, with approximately 15% (PAM 30 s) and 22% (PAM 60 s) higher toxicity than MCF-7. In the case of the MCF-7 cell line, at 24 h the cytotoxic effect of PAM 60 s was more rapid than PAM 30 s, while no significant differences were registered for MDA-MB-231. In the PAM and PTX combined treatment, for MCF-7 cells at the 24 h point, PAM 30 s was able to significantly decrease cell viability, improving the PTX cytotoxicity regardless of the administrated concentration. The cell viability was decreased through the association with PAM 30 s for all three tested concentrations: at 0.1 µM PTX from 71.17% (PTX alone) to 65.99% (PTX and PAM 30 s); at 0.01 µM PTX from 83.55% (PTX alone) to 54.95% (PTX and PAM 30 s); and at 0.001 µM PTX from 100% (PTX alone) to 48.64% (PTX and PAM 30 s). PTX associated with PAM 30 s seemed to exhibit a higher cytotoxicity administrated in lower doses, the combined treatment with 0.001 µM PTX showed a cell viability of 48.64%, while the 0.1 µM PTX and PAM 30 had a cell viability of 65.99%; these results were unexpected. Furthermore, this higher cytotoxicity tendency on lower PTX dose was rejected by the Tukey multiple comparison test between all three combined treatments, being statistically unsignificant (Figure 2B). PAM 60 s was also able to improve PTX cytotoxicity, significantly decreasing the cell viability to values between 34.23% (0.1 µM/0.001 µM PTX and PAM 60 s) and 35.59% (0.01 µM PTX and PAM 60 s). For the same cell line (MCF-7) at the 48 h point, the cytotoxic effects of the combined treatments (PTX and PAM) were more similar to each other, regardless of both PTX concentration and PAM generation time, the value of which ranged between 33.18% and 39.82%. Similarly, the association of PAM with PTX significantly improved the cytotoxicity of PTX for the MDA-MB-231 cell line regardless of the PTX concentration and PAM generation time, showing values between 33.77% and 36.28% at 24 h, and between 18.80% and 19.95% at 48 h.

Overall, our data clearly showed that PAM has the potential to increase PTX sensitivity to both breast cancer cell lines tested (MCF-7 and MDA-MB-231). In addition, the obtained results showed that between the two PAMs, significant differences were not observed in generation time, as in both cases PAM was able to improve PTX cytotoxicity. Park et al., using cold atmospheric plasma directly targeted to cell cultures, were able to restore the sensitivity to PTX in MCF-7-resistant cancer cells [41]. Likewise, in our experimental model, plasma-activated media have the potential to improve PTX cytotoxicity, being much easier to produce and manipulate than in the case of the direct plasma treatment. 

Cell viability was assessed using the MTT assay at 24 and 48 h after the treatment with PAM/PTX and PAM was applied, and the obtained results show that the plasma-activated media had a significant cytotoxic effect (similar to results presented in [49,50]) and improved PTX efficiency on MCF-7 and MDA-MB-231 cell lines. The media used for activation were serum free, the activated media were immediately added to cell cultures, and the contact time between PAM and cells was 20 min for each experimental group. As other authors noted, the presence of the serum in plasma-activated media plays a protective role to ROS impairment in cancerous cells [51,52]. Therefore, in our experimental conditions, the cells were initially exposed to PAM without serum, and thereafter the PAM was exchanged with fresh media containing serum to allow normal development of the cellular processes. The addition of the complete media after incubation with PAM did not restore the viability of the cancerous cells, as the cell viability continued to further decrease, contrary to some experimental data [53]. The occurrence of the cytotoxic effect was at 24 h after the incubation with PAM, and it may have started a few hours after the exposure, as other experiments highlighted a relevant cytotoxic impact at 12 h from the exposure [53]. Similar reductions of MCF-7 and MDA-MB-231 cell viability (30–40%) were obtained by different research groups, but the majority used direct exposure [54], while the plasma-activated media are gaining popularity around plasma medicine research.

Furthermore, we investigated the effect of PAM 30 s and additionally we choose to reduce the time for PAM generation to 15 s, in order to acquire the effects of a smaller plasma production time. In addition, in previous tests (unpublished data) a range of exposure times (5 s to 90 s) were screened for medium activation by assaying cell viability at 24 and 48 h with the MTT test. Moreover, considering that the 0.1 µM and 0.01 doses of PTX showed similar results in the MTT test, the dose of 0.01 µM of PTX was selected for the next investigations as the component of associative treatment.

### 3.2. Clonogenic Assay

**Figure 3:**
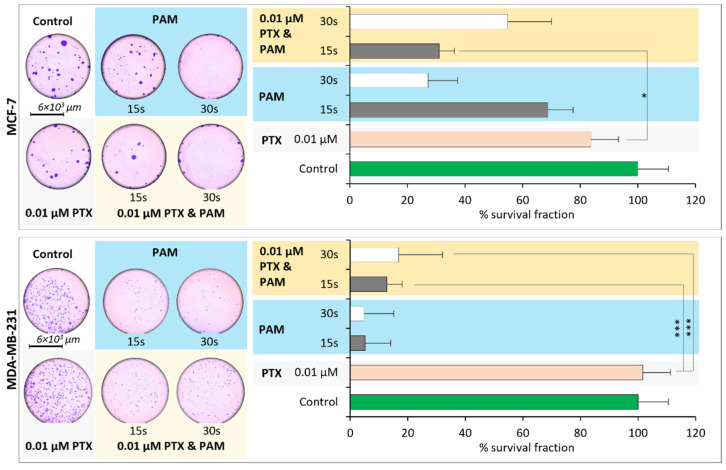
Representative photographs of the MCF-7 and MDA-MB-231 colonies and mean values of the survival fractions as calculated in the case of the control group, PAM 15 s, and PAM 30 s, 0.01 µM PTX, combined treatment (0.01 µM PTX and PAM 15 s and 0.01 µM PTX and PAM 30 s). Data are mean and SEM values (*n* = 3). * *p* < 0.05; *** *p* < 0.001 (ANOVA).

The clonogenic assay was used in order to identify the long-term effects of the different types of treatment on the capacity of the cells to survive, as their capacity to form colonies. Cells were inoculated after the treatment with PTX, PAM, or the combined treatment at 300 cells/well and allowed to grow until colonies developed (50 cells/colony). PE% (plating efficiency) and SF% (survival fraction) were calculated (Figure 3). 

In order to acquire the stability of the effect in time, colony forming assay was performed using PAM 30 s and a lower exposure time for plasma-activated media (PAM 15 s), and for PTX the dose of 0.01 µM was selected. Calculated survival fractions showed that PAM amplified the antiproliferative impact of PTX, but the intensity of the registered effect was different between the two cell lines.

Survival fractions calculated indicated a reduction in the proliferation capacity of MCF-7 cells, the magnitude of the effect being dependent on the type of treatment. Therefore, SF% in the case of PTX was 83.6%, PAM 15 s—68.70%, PAM 30 s—27.10%, while the combined treatment revealed an improvement of PTX efficiency, registering a survival fraction of 30.97% (0.01 µM PTX and PAM 15 s) and 54.84% (0.01 µM PTX and PAM 30 s). In the case of MDA-MB-231, the sensitivity to the treatment was higher, shown by lower survival fractions. The combined treatment reduced proliferative capacity 8x and 6x higher than in the case of PTX alone, respectively. 

The clonogenic assay revealed that treated cells were impaired to recover and restore the proliferation peace, as the number of colonies were smaller than in the case of control group. The combined treatment reduced the colony number to a number similar or even smaller than that specific to PAM or PTX. 

In the case of MCF-7 and MDA-MB-231 cells, the clonogenic assay revealed that the PTX-treated group recovered after the treatment, and the survival fraction was similar to that of the control group. According to calculated survival fractions, MDA-MB-231 cells were more susceptible to the PAM and combined treatment, while MCF-7 cells proved to be more resistant. Even MDA-MB-231 cells were refractory to the PTX treatment, but PAM was able to restore the sensitivity to PTX, with an accentuate reduction in survival fractions (eight times higher for 0.01 µM PTX and PAM 15 s and six times higher for 0.01 µM PTX and PAM 30 s). The clonogenic assay proved that PAM has the capacity to induce a stable cytotoxic effect and also to improve chemosensitivity to PTX. PAM 15 s, obtained by reducing exposure time to half, improved the efficiency of PTX similar to PAM 30 s. Our results were also in good correlation with those reported by other authors [55,56] from the perspective of the effects of PAM alone, but with respect to the effects of the combined treatment our results are original and not signaled elsewhere. 

### 3.3. Viability of Spheroids

Spheroids, by their 3D structure, offer a better evaluation of the impact on the cell viability or cellular processes due to similarities with the architecture of the body tissues and to establishment of some perfusion gradients.

**Figure 4:**
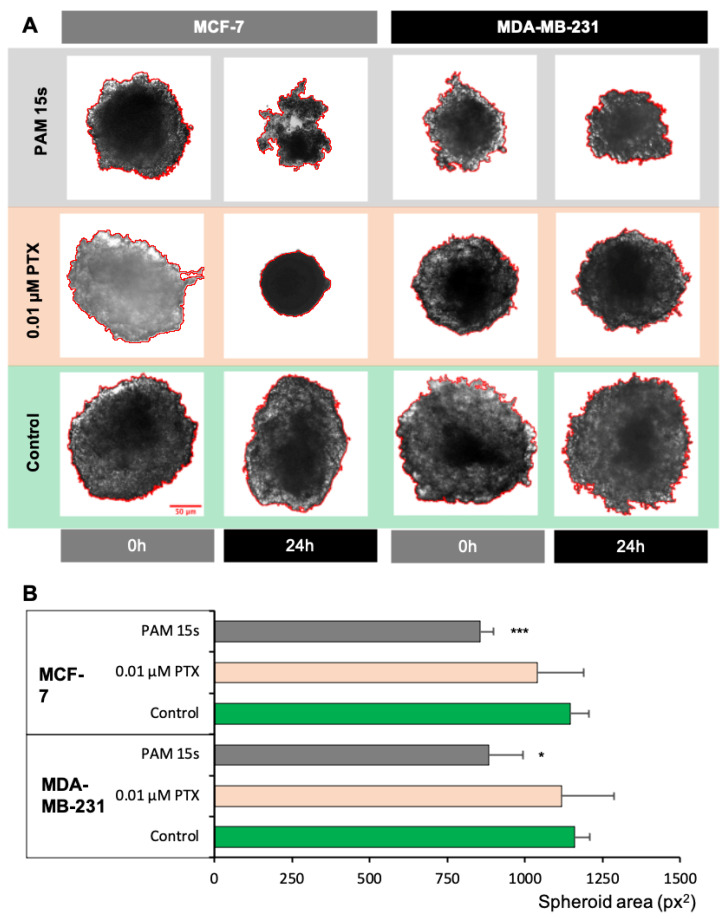
Spheroid area assessment of the MCF-7 and MDA-MB-231 cell culture experimental variants. (**A**) Representative images of the MCF-7 and MDA-MB-231 spheroids obtained by hanging drop method and treated with PTX and PAM 15 s. Column 0 h represents the spheroids before the treatment was added, while column 24 h represents the images of spheroids 24 h after the treatment. The evaluation of shrinkage/growth was assessed by taking photographs of the same spheroid as at the beginning of the treatment. Analysis consisted of the calculation of the area of total spheroid by Fiji software. (**B**) Mean area of spheroids (pixels^2^) following the treatment with PTX and PAM 15 s were acquired at 24 h after the treatment. Data are mean and SEM values (*n* = 8). * *p* < 0.05; ** *p* < 0.01; *** *p* < 0.001 (ANOVA).

Furthermore, we demonstrated on spheroids (Figure 4) generated by the hanging drop method that PAM 15 s is able to infiltrate the 3D structure of MCF-7 spheroids, as it decreased their total area by 25.30% vs. control (*p* < 0.001) and by 17.60% vs. PTX, while in MDA-MB-231 spheroids the shrinkage of the total area was 23.81% vs. control (*p* < 0.05) and 20.95% vs. PTX. As reported in other studies [57], plasma-activated media could interfere with the normal development of cancer spheroids, but due to 3D organization, the effect is smaller than in the case of 2D cell models. One of the solutions is to apply a repeated treatment in order to target sublayers of the spheroid. Here, we also confirmed that even using a shorter time activated medium (PAM 15 s, medium was activated for only 15 s) is capable of inducing a significant cytotoxic effect, as proven by shrinkage of the tumoral spheroids. 

Analysis of the impact on the spheroid development was based on the modifications of the total area of the spheroid as analyzed by the pictures taken before and after treatment. PAM 15 s determined a significant reduction in the spheroid area of both cell lines, with the decreased area suggesting a shrinkage of the spheroid due to loss of cell viability. 

### 3.4. Apoptosis

**Figure 5:**
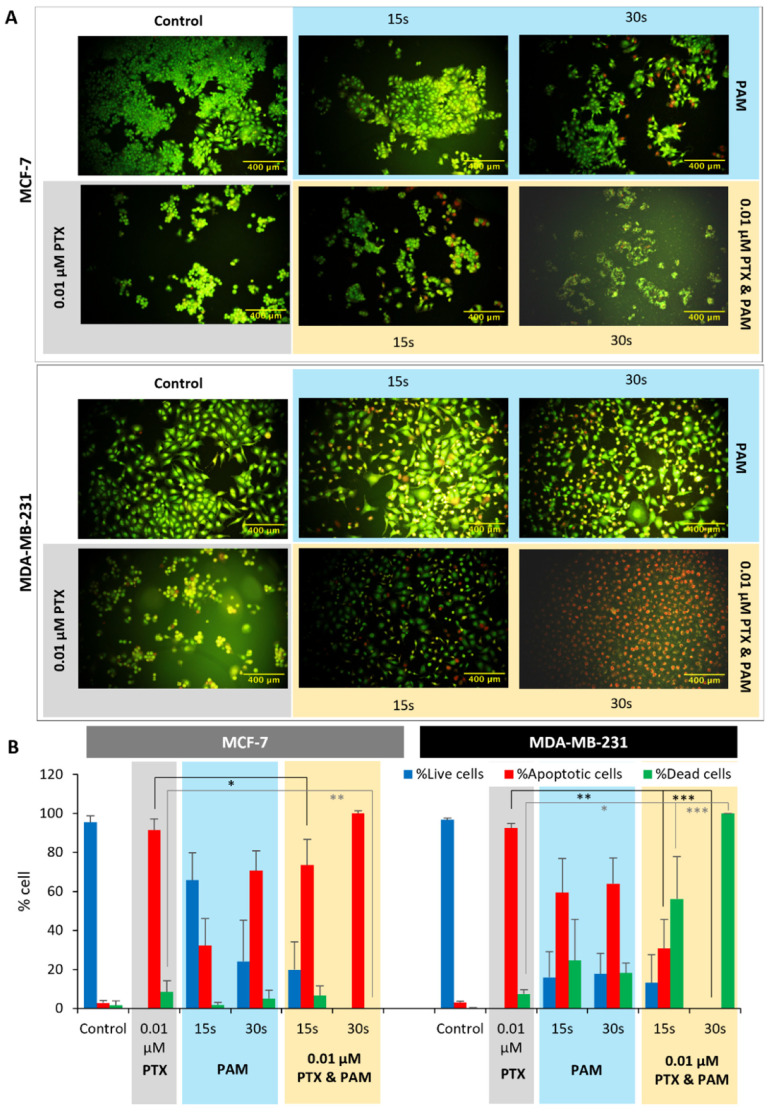
Apoptosis assessment of MCF-7 and MDA-MB-231 cells treated with PAM and PTX. (**A**) Representative images of MCF-7 and MDA-MB-231 under the effect of PTX, combined treatment, and PAM. Untreated cells were cultivated in normal DMEM, without addition of any treatment. PTX-treated cells presented changes in morphology and an increased frequency of apoptotic (condensed green nucleus or orange chromatin) and dead cells. In 0.01 µM PTX and PAM 15 s and 0.01 µM PTX and PAM 30 s groups, the frequency of apoptotic and dead cells significantly increased. PAM 15 s and PAM 30 s showed less frequency of apoptotic and dead cells. (**B**) Distribution of the live, dead, and apoptotic (early and late apoptotic cells) populations (%) of MCF-7 and MDA-MB-231 cell cultures. Data are mean and SEM values (*n* = 3). * *p* < 0.05; ** *p* < 0.01; *** *p* < 0.001 (ANOVA).

The apoptosis of the MCF-7 and MDA-MB-231 cells induced by PTX, combined, or PAM treatments was analyzed using dual staining with AO (acridine orange) and EtBr (ethidium bromide) under fluorescence microscopy 6 h after the treatments (Figure 5). Scoring of the cells was based on the following criteria: live cells—normal green nucleus; apoptotic cells, including early apoptotic cells—bright green nucleus with condensed or fragmented chromatin, and late apoptotic cells—condensed and fragmented orange chromatin; dead cells—structurally normal orange nucleus. Single and combined treatments induced both apoptosis and necrotic death of the cancerous cells. The response of MCF-7 and MDA-MB-231 to the combined treatment exceeded the values of the association members, a balance between necrotic and apoptotic death being observed. 

The MCF-7 cell line was characterized by the presence of a large proportion of apoptotic cells in all experimental groups. In the 0.01 µM PTX and PAM 15 s association, the proportion of apoptotic cells was smaller than in PTX, along with the presence of live cells (19.8%) and dead cells (6.63%). The 0.01 µM PTX and PAM 30 s was able to induce a large proapoptotic effect, the percent of cells in apoptosis being 100%, thereby improving PTX’s effect. Although PAM 15 s seemed to postpone the effect of PTX, the long-term effect was better than PTX 30 s, as proved by the clonogenic assay (30.97% SF for 0.01 µM PTX and PAM 15 s versus 54.84% SF for 0.01 µM PTX and PAM 30 s). It is possible that 0.01 µM PTX and PAM 15 s, with its milder and prolonged effect, resensitized larger populations of cells by gradually diminishing the pool of resistant cells. 

MDA-MB-231 cells were more responsive, and installation of the cytotoxic effect was rapid. If the major effect of PTX was the induction of apoptosis (92% apoptotic cells), 0.01 µM PTX and PAM 15 s rapidly determined apoptosis (30.74% vs. 92% PTX) and subsequently the death (56% vs. 7.40% PTX). In the case of 0.01 µM PTX and PAM 30 s, the effect was faster, as no apoptotic cells were quantified, all being classified as dead (100%). Combining PAM with PTX determined a high diminishing of cell viability, proven also by very low survival fractions in the colony forming assay (12.7% SF 0.01 µM PTX and PAM 15 s and 16.89% SF 0.01 µM PTX and PAM 30 s). 

Estimation of the cell death type was assured by investigation of apoptosis in cancer cell cultures submitted to PAM, PTX, and the combined treatment. Cold atmospheric plasma induces apoptosis in different types of cancerous cells, essentially by inducing DNA damage [58], the main pathway being the mitochondrial one [59,60]. Our experimental results showed that PAM impairs cancer cell viability by the apoptosis mechanism, being in good accordance with other reports [61]. Apoptosis was present in both cell lines, but the reactivity was different and dependent on the type of cell.

### 3.5. Effects of Combined Treatment on Cell Mobility

**Figure 6:**
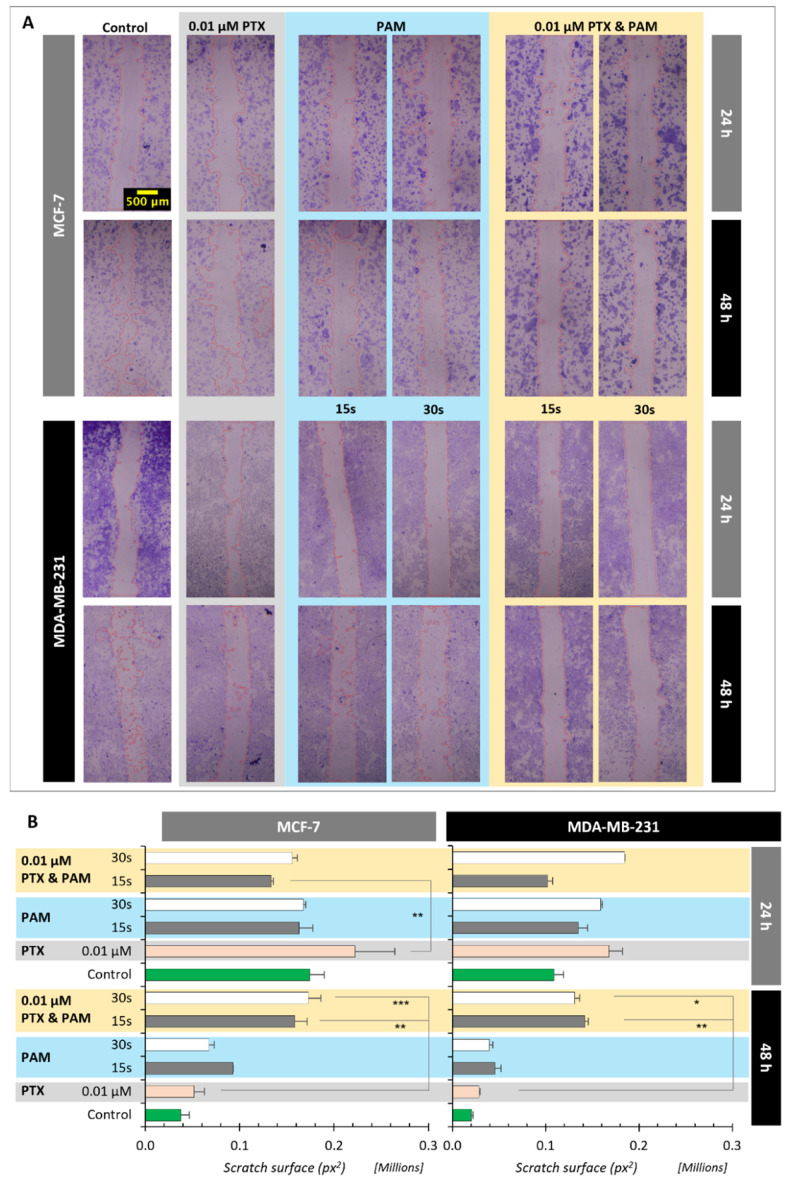
(**A**) Effect of PAM, PTX, and combined treatment on cell migration of MCF-7 and MDA-MB-231 cells, representative images (all images have the same scale as shown in MCF-7 control image, 500 μm). Confluent layers of both cell lines were scratched, and the growth media were exchanged with corresponding media containing PTX and PTX and PAM, while for the control group only fresh complete media were added. Migration of MCF-7 and MDA-MB-231 cells was assessed by taking photos in the same spot at 24 and 48 h (*n* = 3) after the treatment was applied. The baseline was chosen as the initial area delimited by the margins of the scratch, before the treatment was added. The 0.01 µM PTX and PAM 15 s and 0.01 µM PTX and PAM 30 s significantly decreased migration to 48 h (*p* < 0.05, *p* < 0.01). (**B**) The computed results are presented as mean ± SEM values for 3 independent tests (*n* = 3). The statistical significance was established by comparative analysis of the treated groups against PTX using one-way analysis of variance followed by the Tukey post hoc test. * *p* < 0.05, ** *p* < 0.01, *** *p* < 0.001.

The effects of PAM and the combined treatment on cell mobility were determined using a scratch assay (Figure 6). IlastiK and FIJI software were used to measure the scratch area at 24 and 48 h after the treatment with PAM, PTX, and the combined treatment.

At 48 h, the migration of MCF-7 cells calculated on the basis of the scratch area was diminished by 208% (0.01 µM PTX and PAM 15 s), while in the case of 0.01 µM PTX and PAM 30 s this reduction was of 236% when compared with PTX. Taking into account also the effect of PAM on cell migration, 0.01 µM PTX and PAM 15 s determined a reduction in migration of 10%, while 0.01 µM PTX and PAM 30 s reduced cell migration by 45.35%. For MDA-MB-231, when compared with PTX, the 0.01 µM PTX and PAM 15 s calculated area of scratch was 404%, higher than the scratch area of PTX and 0.01 µM PTX and PAM 30 s at 365%. In addition, the comparison with individual effects of the members of combination revealed a reduction in motility of 93% (0.01 µM PTX and PAM 15 s) and of 94% (0.01 µM PTX and PAM 30 s). Overall, the mobility of the MCF-7 and MDA-MB-231 cells was impaired when combined treatment was applied. Twelve hours after, the surface of scratch was approximately three (MCF-7) and five (MDA-MB-231) times larger than in the case of PTX. 

Combined treatment decreased the mobility of both types of cancerous cells, as proven by the highest surface of non-closed scratch determined at 48 h after the treatment, when compared with the PTX and control groups. The effect on cell migration of the combined treatment is higher than the individual effects on cells, demonstrating a synergistic effect. In addition, this effect is due to the induction of apoptosis and reduction in cell viability. The impairment of cell motility by cold atmospheric plasma alone or by activated fluids was acquired for different types of cancer cells (ovarian, breast cancer, human chondrosarcoma cells), with a significant reduction in the cell motility acquired as was assessed in our experimental setup [50,62,63]. The changes in mobility of cancerous cells could be determined by changes in actin cytoskeleton and by decreased secretion of matrix metallopeptidases [64]. Our study has demonstrated for the first time the effect of a combined treatment (PTX and PAM) on the motility of MCF-7 and MDA-MB-231 cancer cells. 

### 3.6. Effects of Combined Treatment on DNA Integrity

**Figure 7:**
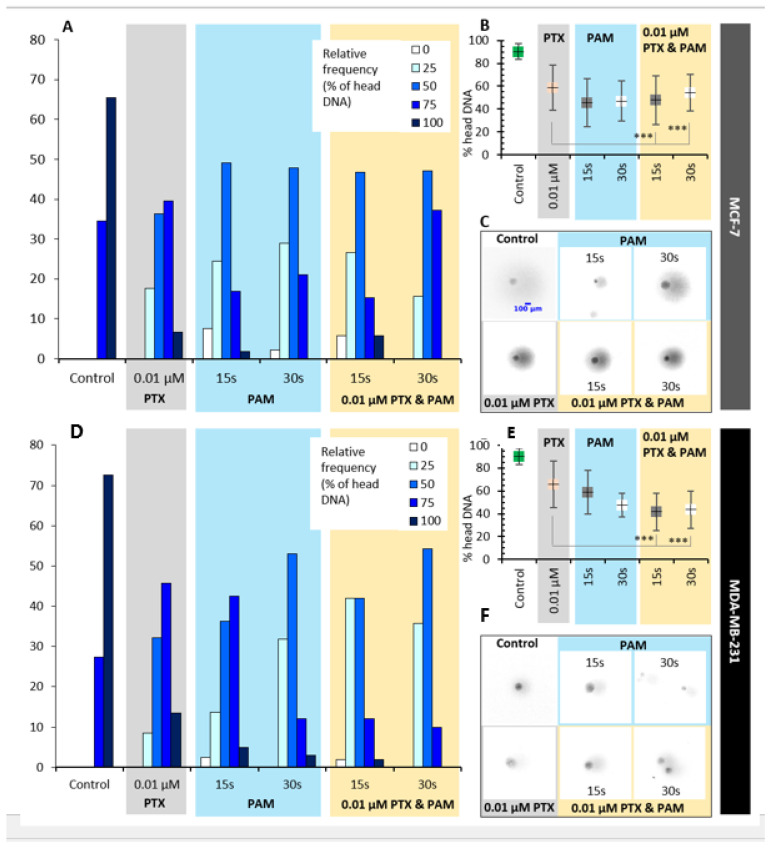
Evaluation of genotoxicity in MCF-7 and MDA-MB-231 cells. (**A**,**D**) Relative frequency of DNA content in experimental groups; (**B**,**E**) content of DNA (%) COMET head; (**C**,**F**) and characteristic microscopic photos of comets, indicating the genotoxic impact of the treatment applied with PTX, PAM, or combined treatment. Data are mean and SEM values (*n* = 3). One-way analysis of variance followed by the Tukey post hoc test was applied in order to evaluate the statistical significance. * *p* < 0.05, ** *p* < 0.01, *** *p* < 0.001.

Impact of the combined treatment on DNA integrity of MCF-7 and MDA-MB-231 cells (Figure 7), as an indicator for genotoxicity, was investigated by single-cell gel electrophoresis, performed in a short-term exposure experiment to catch the early effects on DNA. To depict the consequences of the treatment, the content of DNA (%) and its distribution along different cell populations and experimental conditions were taken into account.

DNA integrity was compromised by the treatment with PTX, PAM, and associated treatment in both MCF-7 and MDA-MB-231 cells, as revealed by a decrease in head DNA content as compared with the control group. In MCF-7, the decrease in head DNA content was of 34.77% for PTX, 49.47% and 47.82% for PAM 15 s and PAM 30 s, respectively, and 46.95% and 40.06% for 0.01 µM PTX and PAM 15 s and 0.01 µM PTX and PAM 30 s, respectively. Both types of PAM amplified the genotoxic effect of PTX; 0.01 µM PTX and PAM 15 s improved the deleterious effect by 18.67%, with 0.01 µM PTX and PAM 30 s by only 8.11%.

Analysis of cell frequency distribution concerning the content of head DNA revealed that over 75% of the cells treated with PTX had DNA content in the range of 35–55%; the cells treated with PAM 15 s were in the 15–65% range, while PAM 30 s was in the 25–75% range. The combined treatment revealed that over 75% of the cells exposed to 0.01 µM PTX and PAM 15 s and 0.01 µM PTX and PAM 30 s fall in 15–75% and 35–75% range of head DNA content, respectively, revealing an improvement in overall PTX’s efficiency mainly by 0.01 µM PTX and PAM 15 s.

In the case of MDA-MB-231 cells, PTX affected DNA integrity by 27.12%; meanwhile, PAM 15 s, PAM 30 s, 0.01 µM PTX and PAM 15 s, and 0.01 µM PTX and PAM 30 s were by 35.00%, 47.34%, 54.02%, and 51.84%, respectively, compared to the control group. As compared with PTX, 0.01 µM PTX and PAM 15 s and 0.01 µM PTX and PAM 30 s improved the genotoxic effect on MDA-MB-231 by 36.91% and 33.92%, respectively. Distribution of cells regarding the content of head DNA revealed that over 75% of the cells treated with PTX had a DNA content in the range of 35–55%, the cells treated with PAM 15 s were in the 55–95% range, and for PAM 30 s were in the 25–65% range. The combined treatment revealed that over 75% of the cells exposed to 0.01 µM PTX and PAM 15 s and 0.01 µM PTX and PAM 30 s fall in 15–55% range of head DNA content, revealing an improvement in overall PTX’s efficiency.

DNA integrity of MCF-7 and MDA-MB-231 cells was acquired by single-cell gel electrophoresis in alkaline conditions, and the content of DNA (%) in head, its distribution along different cell populations, and experimental conditions were quantified. The COMET assay in alkaline conditions, able to detect both single-strand breaks and double-strand breaks, revealed that both types of PAM improved the efficiency of PTX, differentiated between cell lines. While on the MCF-7 line the effect of PAM 15 s was more potent than of PAM 30 s to induce DNA damage compared to the combined treatment, on MDA-MB-231 both types of PAM improved PTX’s efficiency and the sensitivity to combined treatment was higher than in the case of MCF-7 cells. In addition, in our experimental conditions the DNA damage generated by PAM was correlated with a decrease in cell viability, which is in accordance with the findings of Kurita et al. [65].

Breast cancer is a versatile type of cancer and is difficult to treat if not caught in the early stages. Despite advances in optimal management of primary tumors, locoregional relapse occurs in about 10% of patients and can lead to distant metastasis in about 30 to 60% of patients [66]. Systemic treatment, either chemotherapy, hormonal, or immunotherapeutic agents, can be used as adjuvant or neoadjuvant therapy. Unfortunately, after a period of time when the tumor responds to cytotoxic agents, resistant cancer cells are selected, and progression occurs [67]. Although not fully elucidated, some key factors that lead to resistance are: efflux pumps, enzymes that inactivate drugs, resistance genes (e.g., MDR1), cell membrane alterations, DNA repair, and the tumor microenvironment [68]. To overcome some of the challenges regarding relapse and therapy resistance, cold atmospheric plasma is proposed for application in conjunction with chemotherapy.

This is the first research on the effects of plasma-activated media alone or in combination with PTX on MDA-MB-231 and MCF-7 cell lines. We explored whether plasma-activated media could improve the cytotoxic effect of PTX in double- and triple-positive breast cancer cells by associating those two types of treatment. 

Cold atmospheric plasma has been shown to sensitize cancer cells to chemotherapeutic agents in various in vitro studies [69]. Gjika et al. demonstrated that a combined treatment of CAP and temozolomide had a higher geno- and cytotoxic effect on a glioblastoma cell line compared to standalone TMZ therapy [70]. Alimohammadi et al. showed that CAP associated with dacarbazine significantly decreases tumor growth in melanoma B16 cells [71]. 

The present study demonstrated the role of CAP in improving the efficacy of PTX, one of the most used regimes in breast cancer guidelines. The effects of CAP on neoplastic cells are the induction of apoptosis [13,15,18] and cell cycle arrest [20] by generating reactive oxygen or nitrogen species. In addition, an important feature of CAP is its selectivity for neoplastic cells with a reduced damaging impact on normal cells [7,8,10,12]. This trait is a “must have” of oncolytic therapy; the non-selectivity of the current therapeutics and side effects are the major limiting factors to obtain the total efficacy. Here, we presented double- and triple-positive cell lines responsive to combined CAP–PTX therapy in the context of alterations in cell viability, cell mobility, DNA damage, and the capacity of forming colonies. 

In this study, we demonstrated the feasibility of plasma-activated media as an efficient coplayer for PTX in the combined treatment against double- and triple-positive breast cancer cells MCF-7 and MDA-MB-231. In viability studies, the cytotoxicity of PTX was improved by both types of PAM, with no large differences between PAM 30 s and PAM 60 s. We found a beneficial effect of PAM on PTX’s antitumoral effect. This effect was also stable in time, as demonstrated by the clonogenic assay, with a significant decrease in the capacity of colony forming in cells submitted to combined treatment. As apoptosis was investigated, PAM 15 s and PAM 30 s determined apoptosis in MCF-7 and MDA-MB-231 cells, but the level of apoptotic cells was smaller than in the case of PTX. In the combined treatment, PAM accelerated the induction of death in treated cells and assisted in an acceleration of induced death. Our data are in correlation with the data signaled by other authors, even though other authors used plasma jet and not the plasma-activated media [72,73]. As it was correlated with the clonogenic assay, the combined treatment was more efficient that with PTX alone. The clearest proof of a synergistic effect was obtained when cell migration was studied, with delays in scratch closure of approximately three (MCF-7) and five (MDA-MB-231) times more than in the case of PTX. Overall, our data show that PAM and PTX in the combined treatment against double- and triple-positive breast cancer cells MCF-7 and MDA-MB-231 significantly improved the cytotoxic effect of PTX, being a promising treatment in terms of breast cancer therapy. However, additional studies and tests are recommended to make a viable therapy option from the PAM and PTX combined treatment. Even if it is early, we can say that in the future PAM and PTX combined may shift the treatment of breast cancer to a much brighter therapeutic perspective.

## 4. Conclusions

The method to obtain PAM was the dielectric barrier discharge, which is less used in the field of plasma medicine; however, its convenience, simplicity, and reproducibly should be taken into account for future standardization and for obtaining plasma-activated media. 

Our initial results proved the feasibility of PAM to improve PTX’s antitumoral effect in vitro on double- and triple-positive breast cancer cells. We also showed that PAM alone can act as an anticancer agent.

Taking into account the promising anticancer effects and the possibility to associate PAM with PTX, reducing the dose of PTX and subsequently the occurrence of side effects, this method is likely to become a treatment technique in the near future. In addition, the method to obtain PAM is very simple, affordable, can be standardized, and, very importantly, can be administered just like chemotherapy.
